# From Cosmetic Surgery to Critical Care: Clinical Mimicry of Acute Respiratory Distress Syndrome Following Gluteal Augmentation Surgery

**DOI:** 10.7759/cureus.64376

**Published:** 2024-07-11

**Authors:** Ishwari Iyer, Rishav Sinha, Jacqueline Rodriguez, Prahasith Kamani, Nishant Patel, Gaurav S Parhar

**Affiliations:** 1 Internal Medicine, The Brooklyn Hospital Center, Brooklyn, USA; 2 Pulmonary and Critical Care Medicine, The Brooklyn Hospital Center, Brooklyn, USA

**Keywords:** cosmetic procedures, post-operative complication, post-operative pulmonary complications, acute hypoxemic respiratory failure, negative-pressure pulmonary edema, fulminant fat embolism syndrome, pulmonary fat embolism, acute respiratory distress syndrome [ards], brazilian butt lift

## Abstract

Gluteal augmentation surgery, commonly known as the Brazilian Butt Lift (BBL), has become increasingly popular and is offered at numerous surgical centers. Typically performed on an outpatient basis, the procedure takes less than four hours, making it an appealing option for many patients. However, BBL is associated with multiple complications, some of which can be severe, resulting in high mortality rates. Most such post-operative adverse events necessitate urgent transfer to hospitals for optimal care, with post-operative respiratory distress being one such critical sign. Fat embolism syndrome (FES) is a notable complication of BBL. The diagnosis of FES is primarily clinical, supported by imaging studies such as chest X-rays and CT scans. FES often goes underdiagnosed due to the lack of definitive diagnostic criteria and its clinical and radiological similarities to other conditions. Despite its underdiagnosis, FES is reported in approximately 0.06% of patients undergoing BBL. Failure to diagnose it early can lead to complications from empiric treatment of other suspected conditions, potentially worsening the prognosis. Our patient developed respiratory failure within an hour after undergoing BBL. The time to symptom onset and the patient's agitation before the respiratory episode broadened the differential for her condition. This case report highlights the importance of recognizing FES and exploring potential preventive measures, including advancements in surgical techniques and prophylactic strategies.

## Introduction

The Brazilian Butt Lift (BBL) surgery involves the transfer of fat from areas such as the abdomen and thighs to enhance the shape and volume of the gluteal region. In 2016, the average reported mortality rate for patients undergoing BBL surgery was one in every 4,000, with five deaths reported explicitly from South Florida [1]. The complications associated with BBL surgery are not limited to surgical site infection, hematoma and seroma formation, nerve damage, and scarring but also include more severe systemic manifestations such as sepsis, myocardial ischemia, stroke, cardiac arrest, and acute respiratory distress syndrome (ARDS) [2]. Fat embolism syndrome (FES) is a severe complication that affects about one in every 1,473 patients, with a reported mortality rate as high as one in 3,000 [3]. Due to its similarities to other conditions, FES is often misdiagnosed. This case emphasizes the factors contributing to the challenges in diagnosing FES and the importance of recognizing key risk factors for this condition. We further shed light on methods to prevent this potentially life-threatening condition.

## Case presentation

**Figure 1:**
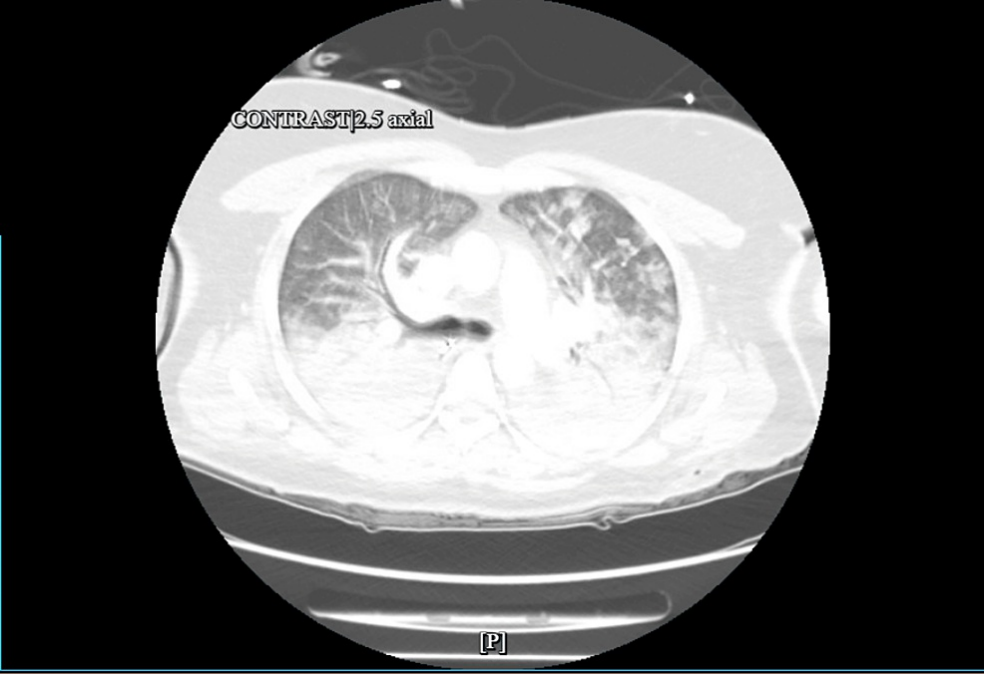
CT Scan on admission demonstrating bilateral ground glass opacities and air bronchograms

**Figure 2:**
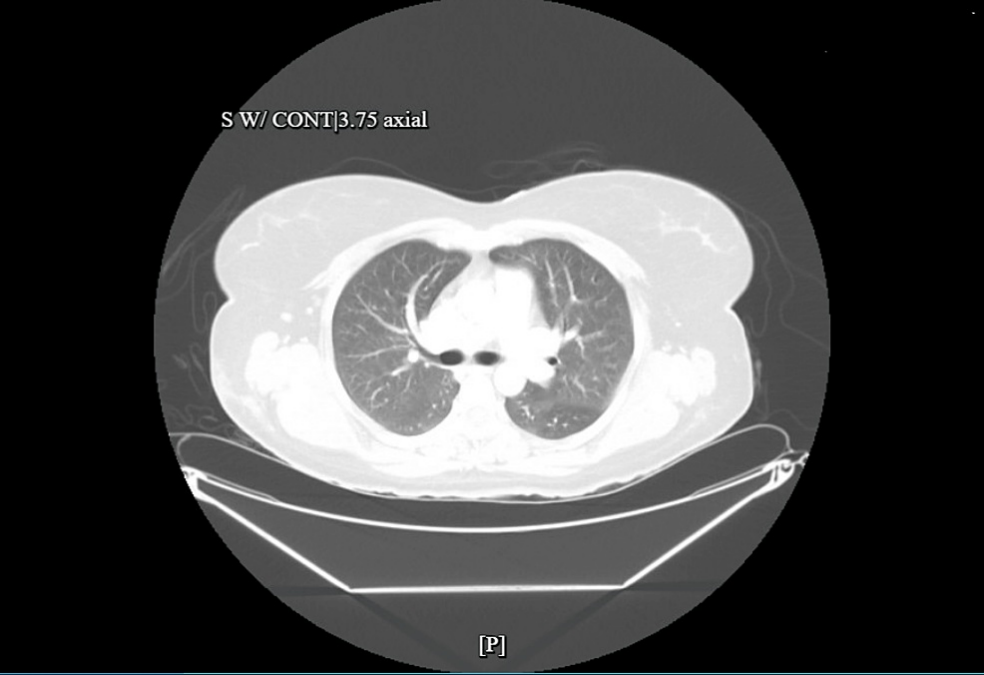
CT scan performed one week after admission demonstrating resolution of opacities previously seen in bilateral lung zones

Our patient is a 47-year-old woman with a history of well-controlled asthma. She was brought to the hospital after developing respiratory distress an hour after BBL surgery at an outpatient surgical facility. The procedure was initially completed without complications. Post-operatively, she was noted to be agitated briefly while on mechanical ventilation; however, she was eventually successfully extubated. An hour after extubation, she developed profound tachypnea with a significant decline in oxygen saturation, dropping to 60%, necessitating re-intubation. There was no stridor or angioedema noted at this time. She received treatment for suspected anaphylaxis before arriving to the emergency room (ER). Upon arrival at the ER, she was found to be hypotensive and had a heart rate of 110 beats per minute, requiring intravenous fluid resuscitation and subsequent vasopressors. She was responsive to pain and followed commands with a Richmond Agitation-Sedation Scale (RASS) score of 0 to -1. A lung exam was significant for coarse rhonchi on bilateral lung zones, with frothy secretions in the endotracheal tube. No edema in the lower extremities, jugular venous distention, petechiae, hives, or calf tenderness was noted. There was no bleeding, erythema, or purulent discharge from the surgical site. Laboratory tests were significant for leukocytosis and lactic acidosis. Arterial blood gas revealed respiratory and metabolic acidosis with a pH of 7.05 and hypoxemia of 115 mmHg on 100% fraction of inhaled oxygen and a positive airway pressure of 5 cm of H2O. High-sensitivity troponin peaked at 4000ng/L. An electrocardiogram showed T-wave inversions in the lateral leads. CT scan of the chest showed extensive bilateral ground-glass opacities with air bronchograms (Figure 1)*. *There were no signs of trauma on abdominal imaging. A CT angiogram of the chest ruled out pulmonary embolism. An echocardiogram revealed normal cardiac function without left atrial enlargement, wall motion abnormalities, or heart strain. She was empirically treated for suspected postoperative aspiration pneumonia with broad-spectrum antibiotics, including coverage for methicillin-resistant Staphylococcus aureus and both typical and atypical organisms. She received 48 hours of heparin infusion for non-ST elevation myocardial infarction (NSTEMI) along with dual antiplatelet and statin, which she tolerated without complications. She continued receiving corticosteroids, antihistamines, and inhaled bronchodilators for suspected anaphylaxis. Within one day, her oxygen requirements were reduced to 40% with resolution of both metabolic and respiratory acidosis. Lung protective ventilation was continued for another day, after which she was extubated. Blood cultures and respiratory cultures were negative. One week later, an interval chest CT showed complete resolution of bilateral infiltrates (Figure 2). She was discharged with outpatient follow-up with cardiology for further ischemia workup.

## Discussion

BBL surgery has become a very common cosmetic procedure in recent years, driven by its accessibility at numerous centers. This increased availability has contributed to its popularity, but it also poses significant risks, especially if postoperative complications arise without adequate support from subspecialties. Postoperative respiratory distress can have various causes. Initially, it is important to consider whether certain medications administered during the procedure may have caused an anaphylactic reaction. This would require immediate treatment with steroids, epinephrine, nebulizers, and possibly reintubation. However, note that the lack of overt airway edema upon re-intubation may either indicate that anaphylaxis has improved with treatment or serve as a signal to investigate other potential contributors to respiratory failure. Inadequate sedation is another cause of postoperative respiratory distress. As seen in our patient, insufficient sedation can lead to agitation and tachypnea due to discomfort. Clenching of the endotracheal tube or airway swelling may lead to an increase in the negative pressure required to overcome this resistance, contributing to a rise in the central venous pressure resulting in pulmonary edema [4]. The presence of bilateral infiltrates on imaging, coupled with leukocytosis, could also occur in aspiration pneumonia. Although aspiration pneumonitis would’ve been a likely cause, the severity of the symptoms and the early need for intubation prompted broad-spectrum antibiotics administration. As time progressed, with rapid improvement in respiratory function and negative microbial cultures, we suspected a non-infectious cause of our patient’s condition.

FES is a rare clinical syndrome that can present with severe respiratory complications as in this case. In this situation, it is presumed that the liposuction may have dislodged lipid particles into the proximal gluteal vessels. This led to fat embolization to the lungs, causing localized inflammation and platelet aggregation, leading to pulmonary edema [5]. Clinical signs of FES typically appear within 12 to 72 hours of the triggering event. Although the classic triad of hypoxemia, neurological dysfunction, and petechiae is rare, it is essential to note that there is no specific diagnostic criterion for FES. The Gurd and Wilson criteria may assist in diagnosis [6];^ ^however, it is neither sensitive nor specific for FES [7].^ ^FES can present with prominent CT Chest findings of bilateral ground-glass opacities along with lobular consolidations as in our case, resembling ARDS. Unlike pulmonary thromboembolism, fat emboli affect the small blood vessels of the lungs and may not be visible as filling defects on a CT angiogram of the chest. Therefore, intact blood vessels on CT angiogram should not be used to rule out FES. The non-specific presentation of FES poses a considerable challenge in clinically and radiologically distinguishing it from ARDS, making the diagnosis complex. Free fatty acids in the bloodstream can also lead to cardiac contractile dysfunction, which has been reported as the cause of cardiac arrest in a 29-year-old following liposuction [8], potentially explaining NSTEMI type 2 in our case.

FES is a common differential diagnosis for postoperative hypoxia following orthopedic surgeries, yet it is often overlooked in the context of cosmetic surgeries. FES typically resolves by itself but requires ventilatory and circulatory support during the acute phase. Early identification of FES is crucial, as it can prevent iatrogenic complications such as dehydration, antibiotic resistance and fluid overload, ultimately enhancing patient safety. Studies indicate that prophylactic corticosteroid use can prevent FES in patients with long bone fractures due to their anti-inflammatory effects, although there is not sufficient data supporting this for cosmetic surgeries [9].^ ^Surgeons devised methods of performing this surgery with "subcutaneous-only" method which has been shown to reduce morbidity and mortality in certain studies. Recommendations also include the use of smaller cannula and ultrasound guidance [10]. Further research is necessary to develop safer surgical methods and improve patient outcomes.

## Conclusions

Our case underscores the importance of maintaining a high suspicion for FES in post-BBL patients presenting with respiratory distress and hypoxemia. The diagnostic challenge is compounded by the non-specific nature of the symptoms and the absence of definitive diagnostic tests for FES. Early identification of FES is crucial to prevent empiric treatment of other conditions and reduce the risk of iatrogenic complications. Various techniques have been evolving to reduce the incidence of FES, though their effectiveness, specifically in BBL patients, requires further study. Increased awareness of this complication among healthcare professionals and patients is essential. Educating both groups can lead to more informed decision-making and improved outcomes. Further research into prophylactic measures and advancements in surgical techniques is needed to enhance the safety of BBL procedures. By highlighting the risks and emphasizing the need for vigilance, we aim to contribute to the overall safety and effectiveness of gluteal augmentation surgeries.
